# Analysis of the variation and genetic stability of chloroplast genome of *Pinus taeda*

**DOI:** 10.1186/s12864-025-12504-x

**Published:** 2026-01-27

**Authors:** Ling Wang, Kaibin Jiang, Liangyu Cao, Jiawen Yu, Jimeng Sun, Chunxin Liu, Shaowei Huang, Tianyi Liu

**Affiliations:** 1 College of Forestry and Landscape Architecture, South China Agricultural University, Guangzhou, 510642 China; 2 Guangdong Key Laboratory for Innovative Development and Utilization of Forest Plant Germplasm, Guangzhou, 510642 China

**Keywords:** *Pinus taeda*, Chloroplast genome, SNP, Parent-progeny inheritance, Paternal inheritance

## Abstract

Chloroplasts are utilized in genetic studies due to their compact genomes and simple structure. While their inheritance is predominantly maternal in most plants, it is paternal in many gymnosperms. This study investigated intraspecific variation and inheritance patterns of the chloroplast genome in loblolly pine (*Pinus taeda*). We sequenced chloroplast DNA from 54 individuals in the second generation core breeding population and performed genome-wide SNP discovery via a custom Python pipeline. From the 81 initially identified SNP loci, a final set of 32 high-frequency loci (variation frequency > 5%) was selected for validation, comprising 14 in intergenic spacer regions and 18 within gene regions. Despite overall conservation, we identified a hypervariable region in the *ycf1* gene, which contained 8 SNP loci and accounted for 44.4% of all coding region SNPs. We then designed 23 primer pairs to amplify the regions containing these candidate variants across a 9 × 5 tester design, encompassing a total of 75 individuals (45 progeny and 30 parents). Phylogenetic and haplotype analyses based on these SNPs revealed no maternal haplotype sharing between any of the eight maternal parents and their progeny, and showed that progeny consistently clustered with specific paternal parents, such as W03 and N4. Furthermore, analysis of SNP profiles allowed reconstruction of paternal lineages and detection of patterns indicative of pollen contamination. Collectively, these results validate both the paternal inheritance of the chloroplast genome in *P. taeda* and the potential utility of chloroplast markers in conifer breeding.

**Supplementary Information:**

The online version contains supplementary material available at 10.1186/s12864-025-12504-x.

## Introduction

Chloroplasts are essential organelles in plants, harboring autonomous genetic information and essential genes for photosynthesis, energy metabolism and protein synthesis [1, 2]. The chloroplast genome possesses several intrinsic properties—including its small size, structural stability, and high conservation—that make it particularly suitable for evolutionary studies [3, 4]. These properties are particularly advantageous for phylogenetic reconstruction. For instance, the high conservation of core genes allows for unambiguous alignment across distantly related species, facilitating deep-level evolutionary comparisons. Conversely, the moderately variable non-coding regions (such as intergenic spacers) and certain genes provide resolution for distinguishing between closely related taxa. Compared to the nuclear genome, the chloroplast genome presents additional practical benefits, including a manageable sequence size, ease of sequencing, and strong collinearity due to the rarity of inversions and rearrangements among most land plants [5–8]. This strong collinearity simplifies genome alignment and comparison, while the predominantly uniparental (often maternal) inheritance and lack of recombination present a clear, non-recombining haplotype for tracing maternal lineages. Leveraging these combined features—which offer a unique balance of evolutionary conservation and informative variation—chloroplast genomes have become a powerful and widely used tool for plant phylogenetic studies, species identification, and resolving taxonomic ambiguities [9]. However, it is important to note that the mode of chloroplast inheritance can vary significantly across plant lineages, with gymnosperms like pines presenting a notable exception to the common maternal pattern.

Loblolly pine (*Pinus taeda* L.) is the most commercially significant tree species in the southern United States, attributed to its favorable growth characteristics, desirable wood properties, and extensive distribution range [10]. In addition to serving as an industrial material, *P. taeda* is notable for its rich resin content in all its tissues. The resin’s primary constituents—monoterpenes, sesquiterpenes, and diterpenes—are valued as significant natural resources in the pharmaceutical and chemical industries. These compounds find extensive applications in the production of solvents, coatings, paints, fragrances, rubber, printing inks, and waterproof materials [11, 12]. Given its significant economic value, *P. taeda* has been extensively introduced in China, where it has become the predominant non-native plantation species [13]. Consequently, genetic improvement programs for loblolly pine in China have progressed significantly. Since 2002, structured breeding strategies have been implemented to enhance its productivity and adaptability [14, 15].

Critically for evolutionary studies and breeding applications, chloroplast inheritance in loblolly pine follows a paternal mode, which is common in gymnosperms but contrasts with the maternal inheritance observed in most plants [16–19]. Research into the cytoplasmic genome of pines began in the late 1980s, confirming that pine chloroplasts are paternally inherited [20]. Specifically, the pioneering work by Neale and colleagues (1989) employed Restriction Fragment Length Polymorphism (RFLP) analysis to establish the principle of paternal chloroplast inheritance in loblolly pine through hybrid crosses [21]. While this foundational study conclusively determined the mode of inheritance, the limited resolution of RFLP analysis left unresolved the subsequent question regarding the content of what is inherited. It provided little data on the specific genetic variants (e.g., SNPs, haplotypes) that are paternally transmitted. Consequently, despite the established inheritance mode, a critical gap persists in our understanding of the nature and extent of intraspecific variation within the paternally inherited chloroplast genome of loblolly pine breeding populations. This gap—pertaining to what and how much is inherited, not how—has hindered the development of molecular tools for practical breeding applications [22].

This study had two primary objectives: first, to investigate intraspecific chloroplast genome variation and characterize genetic patterns in loblolly pine through comparative sequencing and SNP analysis; and second, to develop the resulting data into a methodology for kinship identification. The ultimate goal is to apply this tool to overcome persistent challenges in traditional breeding, particularly the labor-intensive process of verifying controlled pollinations.

## Method

### Experimental site and plant materials

All loblolly-pine material was provided by the Yingde Forestry Research Institute, Guangdong Province, P. R. China (24°15′N, 113°25′E). The core breeding population, established in 1996, consists of 68 full-sib families generated from 26 maternal and 30 paternal parents; the present study used a subset of this progeny trial for chloroplast analyses. To discover novel chloroplast SNPs, 54 offspring individuals representing 10 paternal × 13 maternal crosses within the core breeding population were selected for Illumina sequencing (Table S1). Parent–offspring relationships are listed in Table S2.

To characterize inheritance, a second, independent set of 75 samples—also supplied by the same institute—was genotyped; it comprised 45 progenies from a 9 × 5 tester design together with their 30 corresponding parental clones. Among the 30 parents, each of eight maternal genotypes was represented by one ramet (W16 absent), whereas the paternal set consisted of one W03 ramet and seven ramets each of clones N4, S1 and 222; no sample was available for paternal genotype S2 (parent–progeny matches in Table S3, parental identities in Table S4).

### DNA extraction and sequencing

Total DNA from the core breeding population was extracted from fresh needles using the Polysaccharides & Polyphenolics-rich Plant DNA Kit (DP350, TIANGEN, China), following the manufacturer’s instructions. Libraries were prepared in accordance with the manufacturer’s instructions, targeting an average insert size of 350 bp. The cpDNA from 54 individuals was sequenced on the Illumina Hiseq 4000 platform (Illumina, USA) at the Science Corporation of Gene (SCGene) (Guangzhou, China), employing a paired-end 150 bp sequencing approach. Read quality was evaluated using fastp (v 0.23.2), with the window size parameter set to 8 nucleotides and the minimum acceptable Phred score for quality set to 20. Clean data were obtained after the removal of sequencing adapters and primer sequences, as well as the filtering low-quality reads.

### Chloroplast SNP calling and filtering

Clean paired-end reads were aligned to the *P. taeda* chloroplast reference genome (NC_021440.1) [23] using BWA-MEM v0.7.17 with default settings. Per-sample average depth and genome coverage were extracted from the alignments with samtools coverage v1.14 (Supplementary Table S5). Chloroplast SNPs were called by the sequencing provider (SCGene, Guangzhou, China) using a custom Python routine. The analysis utilized uniquely and properly paired reads and applied the following filters: (i) mapping quality ≥ 20, (ii) base quality ≥ 20, (iii) coverage depth ≥ 3×, and (iv) a homoplasmic genotype. For phylogenetic analyses, positions with missing data (‘N’) were treated as identical to the reference state.

### Chloroplast SNP comparison

To validate the putative SNPs, 23 loci with minor allele frequency ≥ 5% (in the 54 re-sequenced offspring) were selected. Primer pairs were designed with Primer3 (v2.3.6) [24], targeting an annealing temperature of 51 °C (Table S6). Target chloroplast fragments were amplified from total DNA of all 45 offspring and their 30 parents, followed by Sanger sequencing. The resulting sequences were aligned using DNAMAN (v 9.0) with the default alignment parameters (Gap Open Penalty = 10, Gap Extension Penalty = 5). validated SNPs showed identical base calls between Illumina inference and Sanger traces with clean single peaks.

### Identification of chloroplast genetic relationship

To determine the phylogenetic placement of the 75 individuals (45 progeny + 30 parents), a concatenated SNP matrix was generated and a maximum-likelihood tree was constructed in MEGA 11 [25–27] under the GTR + G model (5 gamma categories) with 1,000 bootstrap replicates.

## Result

Chloroplast genome variation among 54 offspring of the core breeding population.

Raw reads were deposited in GenBank (PRJNA1205661). Mean sequencing depth was 12.3×, with 98% of the chloroplast genome covered at ≥ 1×. We identified 81 SNP loci, from which a final set of 32 high-frequency, biallelic SNPs was retained for subsequent analysis. Their population frequency distribution was as follows: ten loci were present in 5.6% (3/54) of individuals, four in 18.5% (10/54), three in 7.4% (4/54), and three in 16.7% (9/54). Notably, two loci (45193, 68985) were nearly fixed (98.2%) in the population, representing fixed differences between the breeding germplasm and the reference genome. These data suggest the presence of detectable intraspecific polymorphis in the chloroplast genome of this breeding population (Table 1).

**Table 1:** SNP loci identified in 54 individuals from the Pinus taeda core breeding population This table includes only loci that were variable in three or more individuals. The genotype data are presented as follows: cells contain the alternative base for a polymorphic site, "-" to indicate an exact match to the reference sequence, and "N" for missing data

site^a^	10,348	14,321	23,167	23,593	29,449	37,238	37,239	37,240	45,193	45,592	50,158	50,842	50,999	59,616	65,304	68,985	90,253	95,372	97,585	98,314	101,103	101,121	101,130	101,139	101,148	101,157	106,236	110,347	113,045	118,329	119,153	119,714
Ref ^b^	T	G	G	C	T	T	T	T	G	G	T	C	G	C	C	G	A	G	T	G	T	T	T	G	G	T	G	T	T	C	G	C
Ⅰ-22	-	T	-	-	-	A	A	A	T	-	-	-	-	-	-	T	G	-	-	-	-	-	G	T	T	-	-	-	G	A	-	-
Ⅰ-54	-	-	-	-	-	-	-	-	T	-	-	-	-	-	-	T	-	-	-	-	-	G	G	-	-	-	-	-	-	-	T	-
Ⅰ-56	-	-	A	A	-	-	-	-	T	-	-	T	A	-	-	T	-	-	-	-	G	G	-	T	-	-	-	-	-	N	-	N
Ⅰ-58	-	-	-	A	-	-	-	-	T	-	-	T	N	-	-	T	-	-	-	-	G	-	-	T	-	-	-	-	-	-	N	-
Ⅱ-12	-	-	-	N	-	-	-	-	T	-	-	-	-	-	-	T	G	-	-	-	-	-	-	-	-	-	-	N	-	-	N	-
Ⅱ-16-4	-	-	-	-	-	-	-	-	T	-	-	-	-	-	-	T	-	-	-	-	G	-	-	-	-	-	-	-	-	-	T	T
Ⅱ-16-5	-	-	A	A	-	A	A	A	T	-	-	T	A	-	-	T	-	-	-	-	G	G	-	-	-	-	-	-	-	-	-	-
Ⅱ-19	A	-	-	-	-	-	-	-	T	-	-	-	-	T	-	T	-	-	-	-	-	-	-	-	-	G	-	-	-	-	T	-
Ⅱ-31-5	-	-	-	A	-	A	A	A	T	-	C	T	A	-	-	T	-	-	-	-	G	-	-	T	-	-	A	-	-	-	-	-
Ⅱ-31-6	-	-	-	-	-	A	A	A	T	-	-	-	-	-	-	T	-	-	-	-	-	-	-	-	-	-	-	-	-	-	-	-
Ⅱ-4-1	-	-	-	-	-	-	-	-	T	T	-	-	-	-	-	T	-	-	-	-	-	-	-	-	-	-	-	-	-	-	-	-
Ⅱ-4-5	-	-	-	-	-	-	-	-	T	T	-	-	-	-	-	T	-	-	-	-	-	-	-	-	-	-	-	-	-	N	T	-
Ⅱ-4-6	-	-	-	-	-	-	-	-	T	-	-	-	-	-	-	T	G	-	-	-	-	-	-	-	-	-	-	-	-	-	T	-
Ⅱ-51-6	A	-	-	-	-	-	-	-	T	-	-	-	-	-	-	T	-	-	-	T	G	-	-	T	-	-	-	-	-	-	N	-
Ⅱ-55-2	-	-	-	-	N	N	N	N	T	-	-	-	-	-	-	T	G	-	-	-	-	G	-	T	-	-	-	-	-	N	-	-
Ⅱ-57-2	-	-	-	-	-	-	-	-	T	-	-	-	-	-	-	T	-	-	-	-	-	-	-	-	-	G	-	-	-	-	-	-
Ⅱ-57-5	-	-	-	-	-	-	-	-	T	-	-	-	-	-	-	T	-	-	-	-	-	-	-	T	T	-	-	-	-	-	-	T
Ⅱ-6-2	-	T	-	-	-	A	A	A	T	-	-	-	-	-	-	T	-	-	-	-	-	-	G	T	T	-	-	-	G	A	-	-
Ⅱ-6-6	-	-	A	A	-	-	-	-	T	-	-	T	A	-	-	T	-	-	-	-	G	G	-	T	T	-	-	-	N	-	-	-
Ⅲ-11	-	-	-	-	-	-	-	-	T	-	-	-	-	-	A	T	-	A	G	-	-	-	-	T	-	-	-	-	-	-	-	-
Ⅲ-16-1	-	-	-	-	-	-	-	-	T	-	-	-	-	-	-	T	-	-	-	-	-	G	G	-	-	-	-	G	-	-	-	-
Ⅲ-16-4	-	-	N	-	-	-	-	-	T	-	-	-	-	-	-	T	-	-	-	-	N	G	G	-	-	-	-	G	N	N	-	-
Ⅲ-16-6	-	-	-	N	-	-	-	-	T	-	-	-	-	-	-	T	G	-	-	-	-	G	G	-	-	-	-	-	-	-	T	-
Ⅲ-19	-	-	-	-	-	-	-	-	T	-	-	-	-	-	-	T	-	-	-	-	G	-	-	-	-	-	-	-	-	-	T	-
Ⅲ-20	-	-	-	-	-	-	-	-	T	-	-	-	-	-	-	T	-	-	-	-	G	-	-	-	T	-	-	-	-	-	T	-
Ⅲ-4-1	-	-	-	-	-	-	-	-	T	-	-	-	-	-	-	T	-	-	-	-	-	-	-	-	-	-	-	-	-	-	-	-
Ⅲ-4-4	-	-	-	-	-	-	-	-	T	T	-	-	-	-	-	T	-	-	-	-	-	-	-	-	-	-	-	-	-	N	-	-
Ⅲ-5	-	-	-	-	N	N	N	N	T	-	-	N	N	-	-	N	G	-	-	-	G	-	-	-	-	-	T	N	-	N	N	N
Ⅲ-51-3	-	-	-	-	-	-	-	-	T	-	-	-	-	-	A	T	-	A	G	-	-	-	-	T	-	-	-	-	-	-	T	-
Ⅲ-54-2	-	-	-	A	-	A	A	A	T	-	C	T	A	-	-	T	-	-	-	-	G	-	-	T	-	-	A	-	-	-	-	-
Ⅲ-55	-	-	-	A	-	-	-	-	T	-	-	-	-	-	-	T	-	-	-	-	-	-	G	T	-	-	-	-	-	-	-	-
Ⅲ-56-1	-	-	-	-	-	N	N	N	T	-	-	-	-	-	-	T	-	-	-	T	G	-	G	-	-	-	-	-	-	-	T	-
Ⅲ-64	-	-	-	-	-	-	-	-	T	-	-	-	-	-	-	T	-	-	-	-	-	-	-	T	T	-	-	-	-	-	-	-
Ⅳ-11-3	N	-	-	A	-	A	A	A	T	-	C	T	A	-	-	T	-	-	-	-	G	-	-	T	-	-	A	-	-	-	-	-
Ⅳ-11-5	-	-	-	-	G	-	-	-	T	-	-	-	-	-	-	T	-	-	-	-	-	-	G	-	-	-	-	-	G	-	N	-
Ⅳ-16	-	-	-	A	-	A	A	A	T	-	C	T	A	-	-	T	G	-	-	-	G	-	-	T	-	-	A	-	-	N	-	-
Ⅳ-17-1	-	-	-	-	-	-	-	-	N	N	-	-	-	T	-	T	-	-	N	-	-	-	-	-	-	G	-	-	-	N	N	-
Ⅳ-17-4	-	-	-	-	-	-	-	-	T	-	-	-	-	-	-	T	-	-	-	-	G	-	-	-	-	-	-	-	-	-	T	-
Ⅳ-19	A	-	-	-	-	-	-	-	T	-	-	-	-	T	-	T	-	-	-	-	-	-	-	-	-	G	-	-	-	-	N	A
Ⅳ-19-4	-	-	-	-	-	-	-	-	T	-	-	-	-	-	A	T	-	A	G	-	-	-	-	T	-	-	-	-	-	-	-	-
Ⅳ-40-2	-	-	-	-	-	-	-	-	T	-	-	T	A	-	-	T	-	-	-	-	-	-	-	-	-	-	-	-	-	-	-	-
Ⅳ-40-5	N	-	-	-	-	-	-	-	T	-	-	T	A	-	-	T	-	-	-	-	-	-	G	T	-	-	-	-	-	-	-	-
Ⅳ-55	-	-	-	-	G	-	-	-	T	-	-	-	-	-	-	T	-	-	-	-	-	-	G	-	-	-	-	-	G	-	-	-
Ⅳ-57	-	-	-	-	-	-	-	-	T	-	-	-	-	-	-	T	-	-	-	-	-	G	G	-	-	-	-	G	-	-	-	-
Ⅳ-58-4	-	-	-	-	-	-	-	-	T	-	-	-	-	-	-	T	-	-	-	-	G	-	-	T	-	-	-	-	-	-	-	-
Ⅴ-1	-	T	-	-	-	A	A	A	T	-	-	-	-	-	-	T	-	-	-	-	-	-	G	T	T	-	-	-	G	A	-	-
Ⅴ-14	-	-	-	-	-	-	-	-	T	-	-	-	-	-	-	T	-	-	-	-	G	-	-	-	T	-	-	-	-	-	T	-
Ⅵ-11	-	-	-	-	-	-	A	A	T	-	-	N	-	-	-	T	-	-	-	T	-	-	-	T	-	G	-	-	-	N	T	-
Ⅵ-12	N	-	-	-	-	N	N	N	T	-	-	-	-	-	-	T	-	-	-	-	N	N	N	N	-	-	-	-	-	-	N	N
Ⅵ-14	-	-	-	-	-	-	-	-	T	-	-	-	-	-	-	T	-	-	-	-	G	-	-	-	-	-	-	-	-	-	T	T
Ⅵ-16	-	-	-	-	-	-	-	-	T	-	-	-	-	-	-	T	-	-	-	-	-	G	G	-	-	-	-	G	-	-	-	-
Ⅵ-19	-	-	-	-	-	-	-	-	T	-	-	-	-	-	-	T	-	-	-	-	-	-	-	T	-	G	-	-	-	N	N	N
Ⅵ-58	-	-	-	-	-	-	-	-	T	-	-	-	-	-	-	T	-	-	-	T	G	-	-	T	-	-	-	-	-	N	T	-
Ⅵ-58-6	-	-	-	-	G	-	-	-	T	-	-	-	-	-	-	T	-	-	-	-	G	-	G	-	-	-	-	-	G	-	-	T

^a^The position of the SNP in the chloroplast genome. Physical position on the chloroplast genome

^b^*Pinus taeda* chloroplast reference genome (NC_021440.1)

A total of 32 high-frequency SNP loci (MAF > 5%) were identified within the loblolly pine chloroplast genome and annotated to specific genes or intergenic spacer regions (Table 2). Of these, 14 resided in intergenic spacers, and 18 were located within nine protein-coding or RNA genes. These 18 genic SNPs were distributed across genes encoding a range of functions, including subunits of ATP synthase (*atpH*), RNA polymerase (*rpoC1*), and the ribosome (*rps11*, *rpl2*), as well as in the *ycf1* and *ycf2* genes of unknown function.

Notably, the distribution of genic SNPs was highly uneven. The *ycf2* gene contained three SNPs, representing 16.7% of the genic SNPs. Eight SNP loci were identified in *ycf1*, representing 9.88% of the total 81 SNP loci and accounting for 44.4% of the 18 SNP loci within gene regions.

**Table 2:** Genes or gene intervals of Chloroplast genome SNP loci loci located between two genes (e.g., rpoC1 ~ rpoB) are in intergenic regions. Gene names are italicized

site	Reference base	Mutated base	Gene or interval	Gene products
10,348	T	A	*trnG-GCC*	tRNA-Gly
14,321	G	T	*atpH*	ATP synthase CF0 C subunit
23,167	G	A	*rpoC1*	RNA polymerase beta’ subunit
23,593	C	A	*rpoC1 ~ rpoB*	RNA polymerase beta’ subunit, RNA polymerase beta subunit
29,449	T	G	*psbM ~ trnD-GCA*	photosystem II protein M, tRNA-Asp
37,238	T	A	*psbJ ~ petA*	photosystem II protein J, cytochrome f
37,239	T	A	*psbJ ~ petA*	
37,240	T	A	*psbJ ~ petA*	
45,193	G	T	*rbcL ~ atpB*	ribulose-1,5-bisphosphate carboxylase/oxygenaselarge subunit, ATP synthase CF1 beta subunit
45,592	G	T	*rbcL ~ atpB*	
50,158	T	C	*trnV-UAC ~ trnH-GUG*	tRNA-Val, tRNA-His
50,842	C	T	*trnH-GUG ~ trnT-GGU*	tRNA-His, tRNA-Thr
50,999	G	A	*trnT-GGU ~ rps4*	tRNA-Thr, pseudo
59,616	C	T	*rps11*	ribosomal protein S11
65,304	C	A	*rpl2*	ribosomal protein L2
68,985	G	T	*trnL-UAA ~ trnT-UGU*	tRNA-Leu, tRNA-Thr
90,253	A	G	*rrn23*	23 S ribosomal RNA
95,372	G	A	*chlN*	photochlorophyllide reductase subunit N
97,585	T	G	*ycf1*	hypothetical chloroplast protein
98,314	G	T	*ycf1*	
101,103	T	G	*ycf1*	
101,121	T	G	*ycf1*	
101,130	T	G	*ycf1*	
101,139	G	T	*ycf1*	
101,148	G	T	*ycf1*	
101,157	T	G	*ycf1*	
106,236	G	A	*psaC ~ ccsA*	photosystem I subunit VII, cytochrome c biogenesis protein
110,347	T	G	*trnV-GAC ~ rps7*	tRNA-Val, ribosomal protein S7
113,045	T	G	*rps 7 ~ trnL-CAA*	ribosomal protein S7, tRNA-Leu
118,329	C	A	*ycf2*	hypothetical chloroplast protein
119,153	G	T	*ycf2*	
119,714	C	T	*ycf2*	

**Fig. 1:**
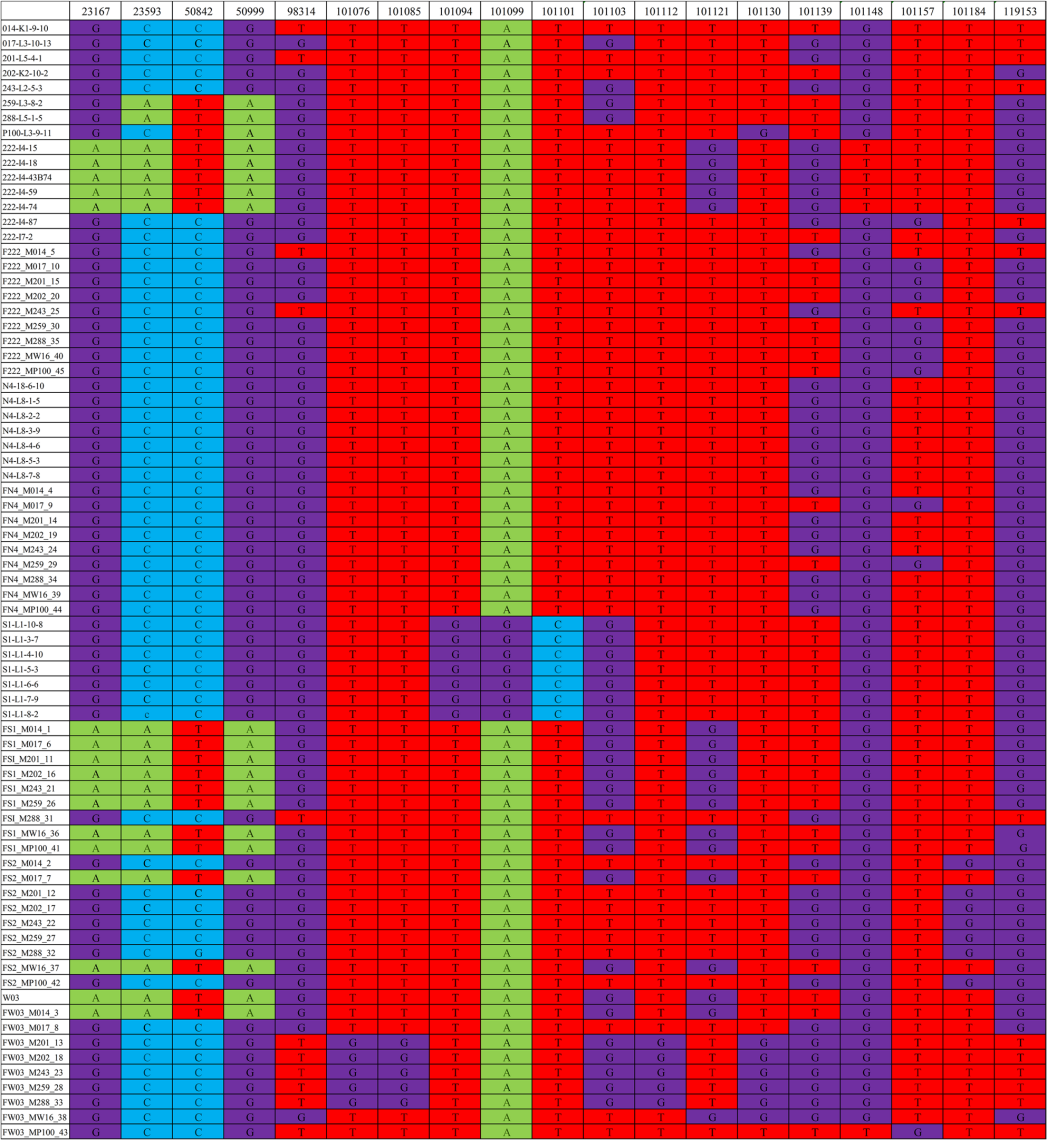
Physical distribution of chloroplast SNPs across 75 *Pinus taeda* individuals. The figure displays the physical positions of identified SNPs (top axis) and the corresponding genotype state for each individual. The individual labels are decoded as follows: “014 K1-9-10” denotes individual 014 from seed orchard location K1-9-10; “F222_M014_5” denotes progeny 5 with paternal (F222) and maternal (M014) parents. Complete coding details are provided in Supplementary Tables S1 and S2. The underlying genotype data are available in Table S7

### Chloroplast SNP validation in 9 × 5 tester progeny

Using 23 chloroplast-specific primer pairs, all 75 samples were successfully amplified and Sanger-sequenced. Subsequent sequence comparison via a custom Python pipeline identified and mapped 19 SNP loci, as shown in Fig. 1.

### Paternal inheritance of chloroplast genome in loblolly pine

The inheritance pattern of the chloroplast genome was analyzed by comparing the haplotypes of 45 progeny with those of their 4 paternal and 8 maternal parents in the tester design.

### Comparison of maternal and offspring chloroplast haplotypes

**Fig. 2:**
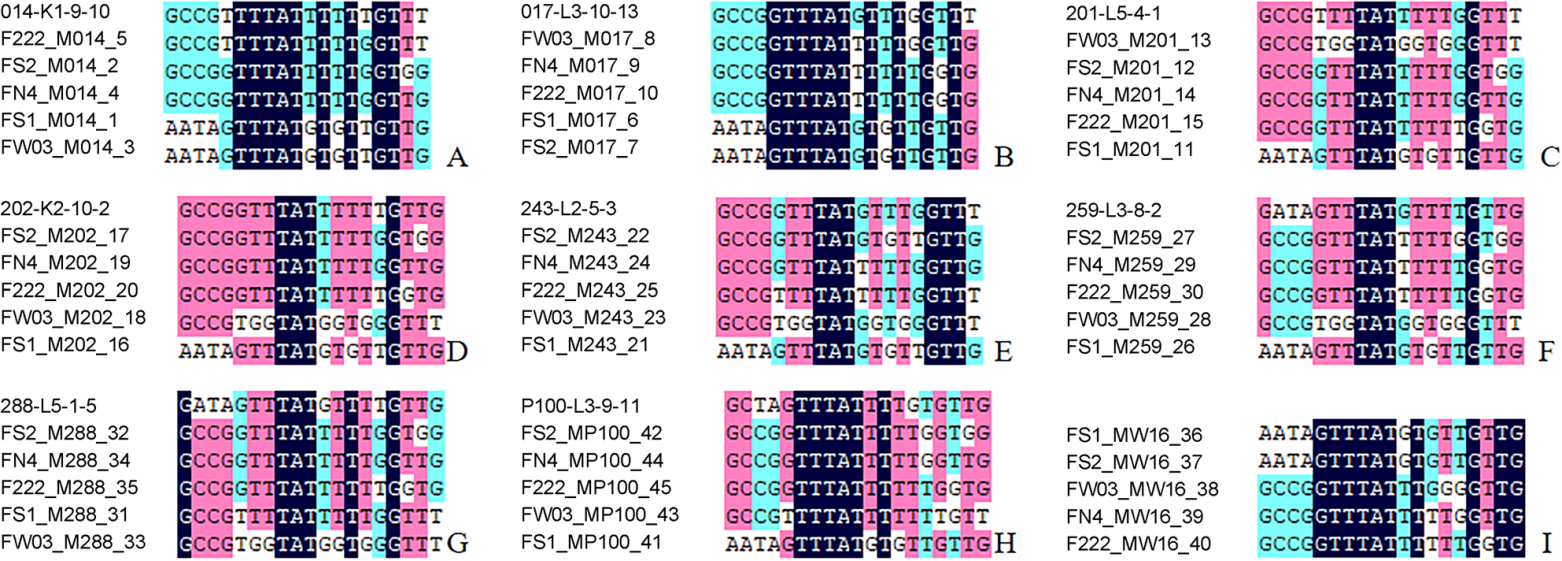
Comparison of chloroplast haplotypes between maternal parents and their offspring Panels **A**-**H** compare the chloroplast haplotypes of eight maternal parents (014, 017, 201, 202, 243, 259, 288, P100) with those of their respective offspring. The analysis shows that the offspring haplotypes are distinct from their corresponding maternal parent in each case. In Panel **I**, the maternal parent (W16) was not sequenced, and SNPs were compared between the offspring. The underlying genotype data for this figure are available in Table S7

The chloroplast SNP profiles of offspring were compared with those of their maternal parents, as summarized in Fig. 2. In each case shown (Fig. 2A and H), none of the progeny shared an identical chloroplast haplotype with their respective maternal parent. In the case where the maternal parent (W16) was unavailable for analysis (Fig. 2I), the SNP profiles were compared exclusively among the offspring. The consistent lack of identical maternal-progeny haplotypes provides the primary genetic evidence against maternal inheritance of the chloroplast in this population.

### Comparison of paternal and offspring chloroplast haplotypes

**Fig. 3:**
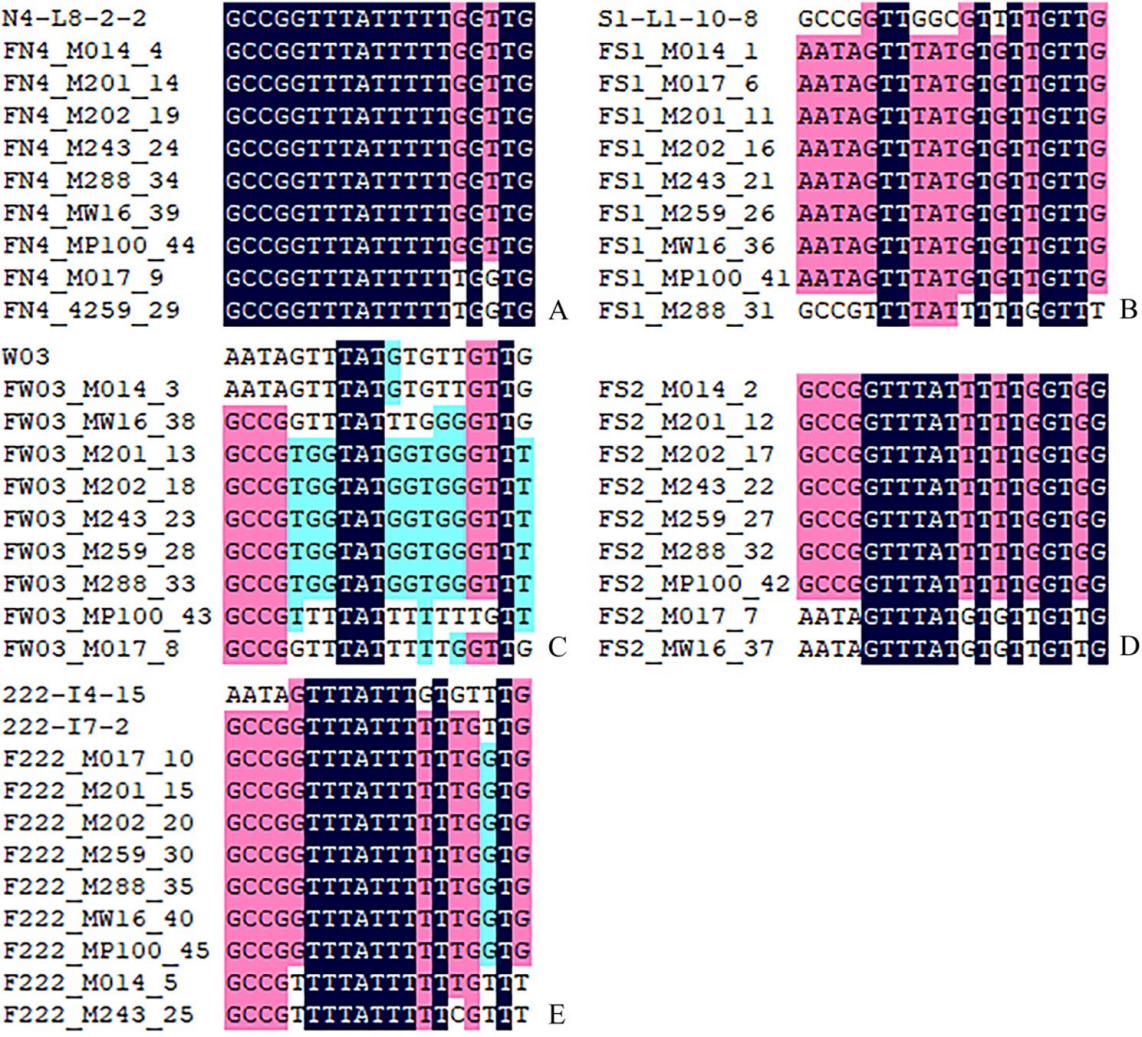
Comparison of chloroplast SNPs between paternal parents and offspring. Panels **A**, **B**, **C**, and **E** show the comparison for paternal parents N4, S1, W03, and 222-I7-2 with their respective offspring. Panel D shows the comparison among the offspring of paternal parent S2, which was not sequenced. The underlying genotype data for this figure are available in Table S7

Analysis of 19 chloroplast SNP loci was conducted across five paternal families (Fig. 3). In the family of paternal parent N4, the chloroplast haplotype was observed to be consistent with those of its seven profiled progeny (4, 14, 19, 24, 34, 39, 44). In the case of paternal parent S1, the haplotype of its eight progeny (1, 6, 11, 16, 21, 26, 36, 41) was found to be inconsistent with S1 but matched that of paternal parent W03; this observation was noted alongside the match between W03 and its own progeny (individual 3). Within the 222 family, progeny haplotypes were consistent with the parent 222-I7-2 at all compared sites except 101,157, where they differed from the sequence of 222-I4-15. For paternal parent S2, where no sample was available, the nine progeny were found to share a highly consistent haplotype among themselves. These observations are consistent with a paternal inheritance mode for the chloroplast genome. These results support that the chloroplast genome is inherited in a paternal manner, while also revealing specific cases of unexpected haplotype distributions.

### Phylogenetic analysis of chloroplast SNPs suggests paternal inheritance

**Fig. 4:**
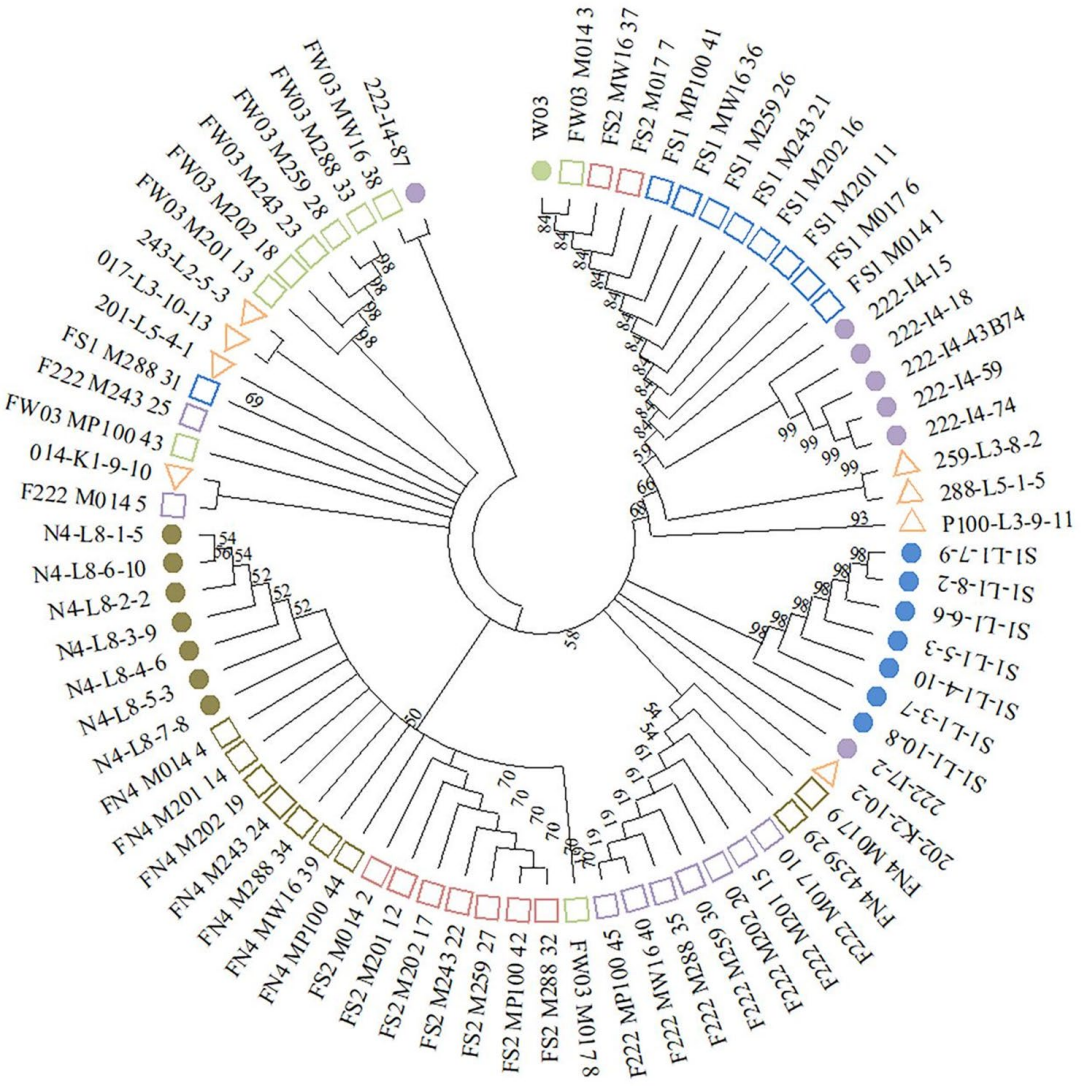
Circular phylogenetic tree of *Pinus taeda* based on 19 chloroplast SNP loci Numbers at the branches represent bootstrap support values. The colors and shapes next to the sample labels distinguish different individuals, which include both parental and offspring samples

A maximum-likelihood tree was reconstructed from the concatenated chloroplast SNP matrix of all 75 individuals (45 progeny + 30 parental ramets) under the GTR + G model with 1,000 bootstrap replicates (Fig. 4). The overall topology showed no maternal haplotype matches: although the eight maternal parents each carried a distinct chloroplast haplotype, none of their corresponding progeny shared the maternal genotype, providing the first line of evidence that the maternal chloroplast genome was not transmitted to the offspring in this cross.

Parent clone W03 and 11 progeny shared the same chloroplast haplotype (bootstrap support = 59%). While the bootstrap value is below the conventional 70% threshold, all 11 offspring exhibited a chloroplast haplotype identical to W03 across the entire 19-SNP matrix (Figs. 3B–D), corroborating W03 as the most probable pollen donor for this cohort.

Nine progeny and the three ramets of clone 222 shared identical haplotypes (bootstrap 68%); 7 progeny and the four ramets of clone N4 shared identical haplotypes (bootstrap 62%); smaller groups of progeny (e.g., 2, 12, 17; 5, 25, 43; 13, 18, 23) shared identical haplotypes with single-ramet paternal genotypes or with each other, indicating further multiple paternal lineages.

Taken together, the observed pattern—an absence of maternal haplotype matches alongside distinct paternal haplotype groups—suggests a predominantly paternal mode of chloroplast transmission within this *P. taeda* tester family and provides tentative indications of the pollen parents involved in the cross.

## Discussion

### Intraspecific variation of the Chloroplast genome in conifers

The loblolly pine chloroplast genome, like other conifer species and most higher plants, shows high structural and gene-order conservation [28–30]. Consistent with this expectation, targeted resequencing of 54 individuals revealed a population-level polymorphism density of 6.7 × 10⁻⁴ per bp (81 SNP loci), aligning with the exceptionally low mutation rate previously reported for conifers.

Complete chloroplast genome survey provides a different perspective on intraspecific diversity compared with fragment-based studies. For example, gene-targeted or small-sample surveys in angiosperms such as Scutellaria baicalensis (25 SNPs across two individuals [31]) and Cyclamen persicum (limited intergenic regions [32]) inevitably focus on predefined amplicons and may therefore underestimate overall chloroplast diversity. Similarly, assessments in Utricularia amethystina relied on restricted sequence data or morphological markers [33]. The common strategy of screening known hypervariable regions, as applied in Japanese larch [34] and *Pseudostellaria heterophylla* [35], or of focusing on genes with distinct evolutionary rates [36], is highly efficient for barcoding or phylogeography, yet it leaves variation outside the targeted windows undetected. Our full-cpDNA scan captures a more complete snapshot of chloroplast polymorphism within a single generation of a *P. taeda* breeding population, while still reflecting the characteristically low substitution rate of the Pinaceae.

The substantial variation we detected in loblolly pine therefore primarily demonstrates the power of a comprehensive chloroplast genome approach. The 81 SNPs provide a near-saturated snapshot of common chloroplast variation within this breeding population, contrasting with the partial picture obtained from pre-selected markers. This does not imply that *P. taeda* possesses higher innate diversity than other species, but it does underscore that common chloroplast diversity in conifers is easily underestimated when only traditional, targeted regions are surveyed. Our results highlight the importance of methodological scope in accurately assessing standing genetic variation.

Crucially, the complete chloroplast genome survey provided sufficient resolution for identifying paternal lineages, as detailed in the following section.

### Paternal inheritance and the interpretation of parent-offspring discrepancies

The mode of chloroplast inheritance in loblolly pine has been previously described as paternal [21]. In the present 9 × 5 tester family, we observed patterns consistent with this model: maternal clones did not share haplotypes with their offspring, while several progeny groups carried chloroplast haplotypes specific to paternal parents (Figs. 2, 3 and 4). These findings suggest the utility of chloroplast SNPs for parentage analysis within breeding populations of this species, even as other studies have noted limitations of chloroplast DNA for delineating species boundaries in certain gymnosperms [37, 38].

Nonetheless, our data included instances where the chloroplast haplotypes of parents and offspring did not match as expected. In evaluating these discrepancies, we considered several biological explanations.

The possibility that de novo mutations created the observed haplotype differences appears low. With a synonymous substitution rate of ~ 0.2 × 10⁻⁹ site⁻¹ year⁻¹ for pine chloroplast DNA [39–42], we expect < 0.02 new mutations per 121 kb haplotype per generation; thus, three or more de novo SNPs within one cross would be highly improbable.

We also considered alternative biological mechanisms. These included rare paternal leakage or even recombination, phenomena that have been documented at low frequencies in some plant species [43, 44]. However, for these to explain the clear-cut cases in our data (e.g., the entire S1 progeny group forming a distinct, uniform haplogroup), they would have to affect 9 out of 45 progeny (20%) of the progeny array - a frequency much higher than the ≤ 1% leakage typically reported in conifers [43, 44]making such scenarios unlikely. Furthermore, we found no evidence of heteroplasmy based on clean, single-peak chromatograms (Sanger detection limit ≈ 10–15%); low-level mixed haplotypes below this threshold cannot be ruled out.

After evaluating these biological alternatives, we consider pollen contamination from unsampled males to be the most parsimonious explanation for those progeny whose haplotypes clearly fell outside any assigned paternal clade. Such contamination is a recognised challenge in operational forestry breeding programmes. Together, our findings are consistent with paternal chloroplast inheritance in loblolly pine and document examples of unexpected pollination. These observations provide a first step toward exploring the use of chloroplast haplotypes as an adjunct for monitoring pedigree integrity in breeding populations.

## Conclusion

This study identified 81 chloroplast SNP loci in a loblolly pine breeding population, 32 of which were high-frequency polymorphisms (MAF ≥ 5%), with *ycf1* and *ycf2* displaying the highest SNP densities (8 and 3 loci, respectively). Phylogenetic and haplotype analyses provided clear evidence for paternal chloroplast inheritance within the 9 × 5 tester family, as shown by the separation of maternal parents from progeny and the clustering of offspring into distinct paternal lineages. The observed mismatches in parent–offspring relationships are most parsimoniously explained by pollen contamination. These findings enhance our understanding of chloroplast inheritance in conifers and offer a cpSNP-based tool for tracking paternal lineages in breeding populations. 

## Supplementary Information

Supplementary Material 1. Table S1: The sample number in core breeding population of *P. taeda*: Ⅱ-31-6 represents the sixth tree from 31 genealogy in the second region group and Ⅵ-14 represents the 14 genealogy in the sixth region group.

Supplementary Material 2. Table S2: The relationships between samples and corresponding parental individuals.

Supplementary Material 3. Table S3: Experimental Samples and Corresponding Parents in *Pinus* taeda tester design.

Supplementary Material 4. Table S4: Parent Samples: aS1 L1-3-7 represents sample S1, which is the 7th tree in the 3rd row of Class 1 in the improved seed orchard of Pinus taeda, S1; b222 I4-15 represents sample 222, which is the 15th tree in Class 4 in the improved seed orchard of Pinus taeda, S1, N4, 222 each have 7 ramets, while W03 only has one ramet.

Supplementary Material 5. Table S5: Sequencing depth and coverage of the chloroplast reference genome for each of the 54 individuals.

Supplementary Material 6. Table S6: Amplification Primers for Chloroplast Genomes in *Pinus taeda* tester design.

Supplementary Material 7. Table S7: Chloroplast SNP Genotypes in 75 *Pinus taeda* Individuals.

## Data Availability

Sequence data that support the findings of this study have been deposited in NCBI with the primary accession code PRJNA1205661.
