# Supplementary material for: Analysis of the variation and genetic stability of chloroplast genome of Pinus taeda
Source: BMC Genomics. 2026 Jan 27;27:215. doi: 10.1186/s12864-025-12504-x (PMC12917966; doi:10.1186/s12864-025-12504-x)
Supplement: Supplementary file 1 — Supplementary Material 1. Table S1: The sample number in core breeding population of P. taeda: Ⅱ-31-6 represents the sixth tree from 31 genealogy in the second region group and Ⅵ-14 represents the 14 genealogy in the sixth region group. [file 12864_2025_12504_MOESM1_ESM.docx]

**Table S1** The sample number in core breeding population of *pinus taeda*: Ⅱ-31-6 represents the sixth tree from 31 genealogy in the second region group and Ⅵ-14 represents the 14 genealogy in the sixth region group.

| Number | Sample number | Number | Sample number | Number | Sample number |
| --- | --- | --- | --- | --- | --- |
| 1 | Ⅱ-31-6 | 19 | Ⅳ-55 | 37 | Ⅲ-51-3 |
| 2 | Ⅲ-16-6 | 20 | Ⅵ-12 | 38 | Ⅲ-4-4 |
| 3 | Ⅵ-14 | 21 | Ⅱ-6-6 | 39 | Ⅲ-64 |
| 4 | Ⅲ-20 | 22 | Ⅰ-22 | 40 | Ⅵ-11 |
| 5 | Ⅲ-16-1 | 23 | Ⅲ-4-1 | 41 | Ⅵ-16 |
| 6 | Ⅱ-19 | 24 | Ⅱ-16-5 | 42 | Ⅳ-40-5 |
| 7 | Ⅲ-5 | 25 | Ⅵ-58 | 43 | Ⅳ-19 |
| 8 | Ⅱ-4-6 | 26 | Ⅱ-12 | 44 | Ⅳ-17-1 |
| 9 | Ⅰ-54 | 27 | Ⅱ-55-2 | 45 | Ⅵ-19 |
| 10 | Ⅲ-54-2 | 28 | Ⅱ-57-5 | 46 | Ⅳ-11-5 |
| 11 | Ⅳ-40-2 | 29 | Ⅳ-17-4 | 47 | Ⅳ-57 |
| 12 | Ⅲ-11 | 30 | Ⅰ-56 | 48 | Ⅱ-57-2 |
| 13 | Ⅲ-56-1 | 31 | Ⅲ-19 | 49 | Ⅰ-58 |
| 14 | Ⅳ-19-4 | 32 | Ⅱ-16-4 | 50 | Ⅱ-6-2 |
| 15 | Ⅴ-1 | 33 | Ⅲ-16-4 | 51 | Ⅴ-14 |
| 16 | Ⅱ-31-5 | 34 | Ⅱ-4-1 | 52 | Ⅲ-55 |
| 17 | Ⅳ-11-3 | 35 | Ⅵ-58-6 | 53 | Ⅳ-58-4 |
| 18 | Ⅱ-4-5 | 36 | Ⅳ-16 | 54 | Ⅱ-51-6 |
