# Supplementary material for: Analysis of the variation and genetic stability of chloroplast genome of Pinus taeda
Source: BMC Genomics. 2026 Jan 27;27:215. doi: 10.1186/s12864-025-12504-x (PMC12917966; doi:10.1186/s12864-025-12504-x)
Supplement: Supplementary file 2 — Supplementary Material 2. Table S2: The relationships between samples and corresponding parental individuals. [file 12864_2025_12504_MOESM2_ESM.docx]

**Table S2** The relationships between samples and corresponding parental individuals

| Paternal parent | Maternal parent | Genealogy number  of offspring | Paternal parent | Maternal parent | Genealogy number  of offspring |
| --- | --- | --- | --- | --- | --- |
| Q13 | 29 | 6 | 243 | 29 | 4 |
| Q13 | 202 | 12 | 243 | 222 | 14 |
| Q13 | 250 | 18 | 243 | W28 | 55 |
| Q13 | 251 | 22 | 288 | 6 | 1 |
| G10 | 250 | 19 | 288 | 202 | 11 |
| G10 | Q6 | 40 | 288 | 251 | 20 |
| G10 | P43 | 64 | 288 | G01 | 51 |
| G10 | P51 | 67 | 288 | 29 | 5 |
| 24 | 250 | 17 | 13 | 29 | 3 |
| 24 | 279 | 31 | 18 | 222 | 15 |
| 24 | W28 | 54 | 270 | W28 | 56 |
| 24 | P40 | 58 | 289 | 222 | 16 |
|  |  |  | WU32 | W28 | 57 |
