# Supplementary material for: Analysis of the variation and genetic stability of chloroplast genome of Pinus taeda
Source: BMC Genomics. 2026 Jan 27;27:215. doi: 10.1186/s12864-025-12504-x (PMC12917966; doi:10.1186/s12864-025-12504-x)
Supplement: Supplementary file 3 — Supplementary Material 3. Table S3: Experimental Samples and Corresponding Parents in Pinus taeda tester design. [file 12864_2025_12504_MOESM3_ESM.docx]

**Table S3** Experimental Samples and Corresponding Parents in Pinus taeda tester design

| Genealogy | Maternal parent | Paternal parent | Genealogy | Maternal parent | Paternal parent | Genealogy | Maternal parent | Paternal parent |
| --- | --- | --- | --- | --- | --- | --- | --- | --- |
| 15 | 201 | 222 | 14 | 201 | N4 | 12 | 201 | S2 |
| 20 | 202 | 222 | 19 | 202 | N4 | 17 | 202 | S2 |
| 25 | 243 | 222 | 24 | 243 | N4 | 22 | 243 | S2 |
| 30 | 259 | 222 | 29 | 259 | N4 | 27 | 259 | S2 |
| 35 | 288 | 222 | 34 | 288 | N4 | 32 | 288 | S2 |
| 5 | 14 | 222 | 4 | 14 | N4 | 2 | 14 | S2 |
| 10 | 17 | 222 | 9 | 17 | N4 | 7 | 17 | S2 |
| 40 | W16 | 222 | 39 | W16 | N4 | 37 | W16 | S2 |
| 45 | P100 | 222 | 44 | P100 | N4 | 42 | P100 | S2 |
| 13 | 201 | W03 | 11 | 201 | S1 |  |  |  |
| 18 | 202 | W03 | 16 | 202 | S1 |  |  |  |
| 23 | 243 | W03 | 21 | 243 | S1 |  |  |  |
| 28 | 259 | W03 | 26 | 259 | S1 |  |  |  |
| 33 | 288 | W03 | 31 | 288 | S1 |  |  |  |
| 3 | 14 | W03 | 1 | 14 | S1 |  |  |  |
| 8 | 17 | W03 | 6 | 17 | S1 |  |  |  |
| 38 | W16 | W03 | 36 | W16 | S1 |  |  |  |
| 43 | P100 | W03 | 41 | P100 | S1 |  |  |  |
