# Supplementary material for: Analysis of the variation and genetic stability of chloroplast genome of Pinus taeda
Source: BMC Genomics. 2026 Jan 27;27:215. doi: 10.1186/s12864-025-12504-x (PMC12917966; doi:10.1186/s12864-025-12504-x)
Supplement: Supplementary file 4 — Supplementary Material 4. Table S4: Parent Samples: aS1 L1-3-7 represents sample S1, which is the 7th tree in the 3rd row of Class 1 in the improved seed orchard of Pinus taeda, S1; b222 I4-15 represents sample 222, which is the 15th tree in Class 4 in the improved seed orchard of Pinus taeda, S1, N4, 222 each have 7 ramets, while W03 only has one ramet. [file 12864_2025_12504_MOESM4_ESM.docx]

**Table S4** Parent Samples:^a^S1 L1-3-7 represents sample S1, which is the 7th tree in the 3rd row of Class 1 in the improved seed orchard of *Pinus taeda*, S1; ^b^222 I4-15 represents sample 222, which is the 15th tree in Class 4 in the improved seed orchard of *Pinus taeda*, S1, N4, 222 each have 7 ramets, while W03 only has one ramet.

| Paternal parent | Paternal parent | Paternal parent | Maternal parent |
| --- | --- | --- | --- |
| S1 L1-3-7 ^a^ | N4 L8-2-2 | 222 I4-15 ^b^ | 202 K2-10-2 |
| S1 L1-4-10 | N4 L8-5-3 | 222 I4-87 | 288 L5-1-5 |
| S1 L1-10-8 | N4 L8-7-8 | 222 I4-59 | 243 L2-5-3 |
| S1 L1-5-3 | N4 L8-1-5 | 222 I4-43 | 017 L3-10-13 |
| S1 L1-8-2 | N4 L8-4-6 | 222 I4-74 | 014 K1-9-10 |
| S1 L1-6-6 | N4 L8-6-10 | 222 I4-18 | 259 L3-8-2 |
| S1 L1-7-9 | N4 L8-3-9 | 222 I7-2 | 201 L5-4-1 |
|  |  | W03 | P100 L3-9-11 |
