# Supplementary material for: Analysis of the variation and genetic stability of chloroplast genome of Pinus taeda
Source: BMC Genomics. 2026 Jan 27;27:215. doi: 10.1186/s12864-025-12504-x (PMC12917966; doi:10.1186/s12864-025-12504-x)
Supplement: Supplementary file 5 — Supplementary Material 5. Table S5: Sequencing depth and coverage of the chloroplast reference genome for each of the 54 individuals. [file 12864_2025_12504_MOESM5_ESM.docx]

**Table S5** Sequencing depth and coverage of the chloroplast reference genome for each of the 54 individuals

| Sample | Depth | Coverage | Sample | Depth | Coverage |
| --- | --- | --- | --- | --- | --- |
| Ⅰ-22 | 5.08 | 0.94 | Ⅲ-5 | 4.41 | 0.87 |
| Ⅰ-54 | 13.4 | 1 | Ⅲ-51-3 | 6.6 | 0.99 |
| Ⅰ-56 | 8.21 | 0.98 | Ⅲ-54-2 | 8.92 | 0.99 |
| Ⅰ-58 | 8.83 | 0.99 | Ⅲ-55 | 8.58 | 1 |
| Ⅱ-12 | 3.79 | 0.93 | Ⅲ-56-1 | 15.3 | 1 |
| Ⅱ-16-4 | 9.74 | 0.99 | Ⅲ-64 | 15.2 | 0.99 |
| Ⅱ-16-5 | 12.6 | 1 | Ⅳ-11-3 | 11.5 | 0.99 |
| Ⅱ-19 | 16.3 | 1 | Ⅳ-11-5 | 8.72 | 0.99 |
| Ⅱ-31-5 | 25.8 | 1 | Ⅳ-16 | 8.16 | 1 |
| Ⅱ-31-6 | 9.99 | 1 | Ⅳ-17-1 | 3.55 | 0.83 |
| Ⅱ-4-1 | 6.51 | 0.99 | Ⅳ-17-4 | 8.79 | 0.98 |
| Ⅱ-4-5 | 8.28 | 0.99 | Ⅳ-19 | 9.68 | 0.99 |
| Ⅱ-4-6 | 7.58 | 0.99 | Ⅳ-19-4 | 41.1 | 1 |
| Ⅱ-51-6 | 22.6 | 0.99 | Ⅳ-40-2 | 18.3 | 1 |
| Ⅱ-55-2 | 3.97 | 0.93 | Ⅳ-40-5 | 7.92 | 0.98 |
| Ⅱ-57-2 | 31.7 | 1 | Ⅳ-55 | 18.2 | 1 |
| Ⅱ-57-5 | 9.21 | 0.98 | Ⅳ-57 | 25.9 | 1 |
| Ⅱ-6-2 | 10.8 | 0.99 | Ⅳ-58-4 | 8.19 | 0.99 |
| Ⅱ-6-6 | 9.3 | 1 | Ⅴ-1 | 5.81 | 0.97 |
| Ⅲ-11 | 12 | 0.99 | Ⅴ-14 | 8.58 | 0.98 |
| Ⅲ-16-1 | 10.1 | 1 | Ⅵ-11 | 13 | 0.99 |
| Ⅲ-16-4 | 6.17 | 0.99 | Ⅵ-12 | 4.57 | 0.94 |
| Ⅲ-16-6 | 4.82 | 0.97 | Ⅵ-14 | 31 | 1 |
| Ⅲ-19 | 13.8 | 0.99 | Ⅵ-16 | 12.4 | 1 |
| Ⅲ-20 | 30 | 1 | Ⅵ-19 | 8.7 | 0.98 |
| Ⅲ-4-1 | 6.58 | 0.99 | Ⅵ-58 | 7 | 0.98 |
| Ⅲ-4-4 | 15.2 | 0.99 | Ⅵ-58-6 | 23.1 | 1 |
