# Supplementary material for: Analysis of the variation and genetic stability of chloroplast genome of Pinus taeda
Source: BMC Genomics. 2026 Jan 27;27:215. doi: 10.1186/s12864-025-12504-x (PMC12917966; doi:10.1186/s12864-025-12504-x)
Supplement: Supplementary file 6 — Supplementary Material 6. Table S6: Amplification Primers for Chloroplast Genomes in Pinus taeda tester design. [file 12864_2025_12504_MOESM6_ESM.docx]

**Table S6** Amplification Primers for Chloroplast Genomes in *Pinus taeda* tester design

| Primer | Amplification length(bp) | SNP site | SNP type | Primer sequence（5’-3’） | Annealing  temperature （℃） |
| --- | --- | --- | --- | --- | --- |
| G1 | 720 | 10348 | T/A | TTTGTTTACTTGGGTTATCG | 51 |
|  |  |  |  | ATTAGACGATGGACGCTCTT |  |
| G2 | 394 | 14321 | G/T | ATACTAATGTAATGACGAGGTG | 51 |
|  |  |  |  | AGTTGTTCGGAAGAAATCGT |  |
| G3 | 987 | 23167 | G/A | CCTCTATTCAGGCTAACCCA | 51 |
|  |  | 23593 | C/A | GAAATGGAAGTTTGGGCTCT |  |
| G4 | 560 | 29449 | T/G | CATGACCCTGGAATGATAAG | 51 |
|  |  |  |  | ATCGACCTAGCACTCGCATA |  |
| G5 | 464 | 37238 | T/A | GCCGATACCACTGGAAGGAT | 51 |
|  |  | 37239 | T/A | TTCGCATAATCTTCCATCAG |  |
| G6 | 440 | 45193 | G/T | AGTCCATCGGTCCAAACAGT | 51 |
|  |  |  |  | ACTGAAAGGTTCTCGGGTCA |  |
| G7 | 450 | 45592 | G/T | TATGTATGACGCAACCCAATC | 51 |
|  |  |  |  | AATGTTCCATATTGGGTCGA |  |
| G8 | 720 | 50158 | T/C | CTCCGTCGGTAGATGAGCCT | 51 |
|  |  |  |  | CGTTTGTTCTTATGTGGGTC |  |
| G9 | 712 | 50842 | C/T | CCAAGGCAGTGGATTGTGAA | 51 |
|  |  | 50999 | GA | GGAATGGCTCCATATTCTTA |  |
| G10 | 466 | 59616 | C/T | CATTGCGATGCCTATTGTAT | 51 |
|  |  |  |  | GGTCTTCTGCTGGTGCCTGT |  |
| G11 | 660 | 65304 | C/A | GAGAAGGAGCATCTATTGTATT | 51 |
|  |  |  |  | TACAAGAATGGCGATAAGAT |  |
| G12 | 463 | 68985 | G/T | GGACTCTATCTTTATCCTCGTC | 51 |
|  |  |  |  | ATTGGTAGACGCTACGGACT |  |
| G13 | 595 | 90253 | A/G | AAATCAGCGGATGAGTTGTG | 51 |
|  |  |  |  | CCTCCGTGGACGAACCTTGC |  |
| G14 | 499 | 95372 | G/A | GGCAAGAGCAAATGGACTGG | 51 |
|  |  |  |  | CCGTTCCTCTATTTCCTCCA |  |
| G15 | 533 | 97585 | T/G | CGCATACTCAAGTGATGGAA | 51 |
|  |  |  |  | TAAGCGAACGAACAGCATCT |  |
| G16 | 645 | 98314 | G/T | TTCCCACAACTTTCATACCA | 51 |
|  |  |  |  | TTGTCTGCTGGGATAATGGA |  |
| G17 | 579 | 101103 | T/G | AAAATGGTGGAGATGGTGAA | 51 |
|  |  | 101157 | T/G | AGGCCATTAGACTCAGGTCA |  |
| G18 | 534 | 106236 | G/A | AAAGGAGGATAGTCTTCACT | 51 |
|  |  |  |  | ACTGGATCAGGCAGGATAAG |  |
| G19 | 735 | 110347 | T/G | GCTTACATTAGGTTTAGGGATA | 51 |
|  |  |  |  | TCAGCGGGAAGAGGATTGTA |  |
| G20 | 671 | 113045 | T/G | ACTCATAGAATGGCAGAAGC | 51 |
|  |  |  |  | ATCGCACTTCTTCCCTTCAT |  |

**Table S6** Amplification Primers for Chloroplast Genomes in *Pinus taeda* tester design (continued)

| Primer | Amplification length(bp) | SNP site | SNP type | Primer sequence（5’-3’） | Annealing  temperature （℃） |
| --- | --- | --- | --- | --- | --- |
| G21 | 879 | 118329 | C/A | TCGGATAGTAAATCATCTTG | 51 |
|  |  |  |  | ATTTCATGGGCTCCAAAGTA |  |
| G22 | 558 | 119153 | G/T | GTCTGATTGGACCATTTGTA | 51 |
|  |  |  |  | AATAACTAAGCCTAGTCAGG |  |
| G23 | 656 | 119714 | C/T | TGAGTAGGACCCATAAAGAT | 51 |
|  |  |  |  | AATAGAATCGGATTTGTCCT |  |
