# Supplementary material for: Analysis of the variation and genetic stability of chloroplast genome of Pinus taeda
Source: BMC Genomics. 2026 Jan 27;27:215. doi: 10.1186/s12864-025-12504-x (PMC12917966; doi:10.1186/s12864-025-12504-x)
Supplement: Supplementary file 7 — Supplementary Material 7. Table S7: Chloroplast SNP Genotypes in 75 Pinus taeda Individuals. [file 12864_2025_12504_MOESM7_ESM.docx]

**Table S7** Chloroplast SNP Genotypes in 75 *Pinus taeda* Individuals.

| **Sample** | **23167** | **23593** | **50842** | **50999** | **98314** | **101076** | **101085** | **101094** | **101099** | **101101** | **101103** | **101112** | **101121** | **101130** | **101139** | **101148** | **101157** | **101184** | **119153** |
| --- | --- | --- | --- | --- | --- | --- | --- | --- | --- | --- | --- | --- | --- | --- | --- | --- | --- | --- | --- |
| 014-K1-9-10 | G | C | C | G | T | T | T | T | A | T | T | T | T | T | T | G | T | T | T |
| 017-L3-10-13 | G | C | C | G | G | T | T | T | A | T | G | T | T | T | G | G | T | T | T |
| 201-L5-4-1 | G | C | C | G | T | T | T | T | A | T | T | T | T | T | G | G | T | T | T |
| 202-K2-10-2 | G | C | C | G | G | T | T | T | A | T | T | T | T | T | T | G | T | T | G |
| 243-L2-5-3 | G | C | C | G | G | T | T | T | A | T | G | T | T | T | G | G | T | T | T |
| 259-L3-8-2 | G | A | T | A | G | T | T | T | A | T | G | T | T | T | T | G | T | T | G |
| 288-L5-1-5 | G | A | T | A | G | T | T | T | A | T | G | T | T | T | T | G | T | T | G |
| P100-L3-9-11 | G | C | T | A | G | T | T | T | A | T | T | T | T | G | T | G | T | T | G |
| 222-I4-15 | A | A | T | A | G | T | T | T | A | T | T | T | G | T | G | T | T | T | G |
| 222-I4-18 | A | A | T | A | G | T | T | T | A | T | T | T | G | T | G | T | T | T | G |
| 222-I4-43B74 | A | A | T | A | G | T | T | T | A | T | T | T | G | T | G | T | T | T | G |
| 222-I4-59 | A | A | T | A | G | T | T | T | A | T | T | T | G | T | G | T | T | T | G |
| 222-I4-74 | A | A | T | A | G | T | T | T | A | T | T | T | G | T | G | T | T | T | G |
| 222-I4-87 | G | C | C | G | G | T | T | T | A | T | T | T | T | T | G | G | G | T | T |
| 222-I7-2 | G | C | C | G | G | T | T | T | A | T | T | T | T | T | T | G | T | T | G |
| F222_M014_5 | G | C | C | G | T | T | T | T | A | T | T | T | T | T | G | G | T | T | T |
| F222_M017_10 | G | C | C | G | G | T | T | T | A | T | T | T | T | T | T | G | G | T | G |
| F222_M201_15 | G | C | C | G | G | T | T | T | A | T | T | T | T | T | T | G | G | T | G |
| F222_M202_20 | G | C | C | G | G | T | T | T | A | T | T | T | T | T | T | G | G | T | G |
| F222_M243_25 | G | C | C | G | T | T | T | T | A | T | T | T | T | T | G | G | T | T | T |
| F222_M259_30 | G | C | C | G | G | T | T | T | A | T | T | T | T | T | T | G | G | T | G |
| F222_M288_35 | G | C | C | G | G | T | T | T | A | T | T | T | T | T | T | G | G | T | G |
| F222_MW16_40 | G | C | C | G | G | T | T | T | A | T | T | T | T | T | T | G | G | T | G |
| F222_MP100_45 | G | C | C | G | G | T | T | T | A | T | T | T | T | T | T | G | G | T | G |
| N4-18-6-10 | G | C | C | G | G | T | T | T | A | T | T | T | T | T | G | G | T | T | G |
| N4-L8-1-5 | G | C | C | G | G | T | T | T | A | T | T | T | T | T | G | G | T | T | G |
| N4-L8-2-2 | G | C | C | G | G | T | T | T | A | T | T | T | T | T | G | G | T | T | G |
| N4-L8-3-9 | G | C | C | G | G | T | T | T | A | T | T | T | T | T | G | G | T | T | G |
| N4-L8-4-6 | G | C | C | G | G | T | T | T | A | T | T | T | T | T | G | G | T | T | G |
| N4-L8-5-3 | G | C | C | G | G | T | T | T | A | T | T | T | T | T | G | G | T | T | G |
| N4-L8-7-8 | G | C | C | G | G | T | T | T | A | T | T | T | T | T | G | G | T | T | G |

**Table S7** Chloroplast SNP Genotypes in 75 *Pinus taeda* Individuals (continued).

| **Sample** | **23167** | **23593** | **50842** | **50999** | **98314** | **101076** | **101085** | **101094** | **101099** | **101101** | **101103** | **101112** | **101121** | **101130** | **101139** | **101148** | **101157** | **101184** | **119153** |
| --- | --- | --- | --- | --- | --- | --- | --- | --- | --- | --- | --- | --- | --- | --- | --- | --- | --- | --- | --- |
| FN4_M014_4 | G | C | C | G | G | T | T | T | A | T | T | T | T | T | G | G | T | T | G |
| FN4_M017_9 | G | C | C | G | G | T | T | T | A | T | T | T | T | T | T | G | G | T | G |
| FN4_M201_14 | G | C | C | G | G | T | T | T | A | T | T | T | T | T | G | G | T | T | G |
| FN4_M202_19 | G | C | C | G | G | T | T | T | A | T | T | T | T | T | G | G | T | T | G |
| FN4_M243_24 | G | C | C | G | G | T | T | T | A | T | T | T | T | T | G | G | T | T | G |
| FN4_M259_29 | G | C | C | G | G | T | T | T | A | T | T | T | T | T | T | G | G | T | G |
| FN4_M288_34 | G | C | C | G | G | T | T | T | A | T | T | T | T | T | G | G | T | T | G |
| FN4_MW16_39 | G | C | C | G | G | T | T | T | A | T | T | T | T | T | G | G | T | T | G |
| FN4_MP100_44 | G | C | C | G | G | T | T | T | A | T | T | T | T | T | G | G | T | T | G |
| S1-L1-10-8 | G | C | C | G | G | T | T | G | G | C | G | T | T | T | T | G | T | T | G |
| S1-L1-3-7 | G | C | C | G | G | T | T | G | G | C | G | T | T | T | T | G | T | T | G |
| S1-L1-4-10 | G | C | C | G | G | T | T | G | G | C | G | T | T | T | T | G | T | T | G |
| S1-L1-5-3 | G | C | C | G | G | T | T | G | G | C | G | T | T | T | T | G | T | T | G |
| S1-L1-6-6 | G | C | C | G | G | T | T | G | G | C | G | T | T | T | T | G | T | T | G |
| S1-L1-7-9 | G | C | C | G | G | T | T | G | G | C | G | T | T | T | T | G | T | T | G |
| S1-L1-8-2 | G | c | C | G | G | T | T | G | G | C | G | T | T | T | T | G | T | T | G |
| FS1_M014_1 | A | A | T | A | G | T | T | T | A | T | G | T | G | T | T | G | T | T | G |
| FS1_M017_6 | A | A | T | A | G | T | T | T | A | T | G | T | G | T | T | G | T | T | G |
| FSI_M201_11 | A | A | T | A | G | T | T | T | A | T | G | T | G | T | T | G | T | T | G |
| FS1_M202_16 | A | A | T | A | G | T | T | T | A | T | G | T | G | T | T | G | T | T | G |
| FS1_M243_21 | A | A | T | A | G | T | T | T | A | T | G | T | G | T | T | G | T | T | G |
| FS1_M259_26 | A | A | T | A | G | T | T | T | A | T | G | T | G | T | T | G | T | T | G |
| FSI_M288_31 | G | C | C | G | T | T | T | T | A | T | T | T | T | T | G | G | T | T | T |
| FS1_MW16_36 | A | A | T | A | G | T | T | T | A | T | G | T | G | T | T | G | T | T | G |
| FS1_MP100_41 | A | A | T | A | G | T | T | T | A | T | G | T | G | T | T | G | T | T | G |
| FS2_M014_2 | G | C | C | G | G | T | T | T | A | T | T | T | T | T | G | G | T | G | G |
| FS2_M017_7 | A | A | T | A | G | T | T | T | A | T | G | T | G | T | T | G | T | T | G |
| FS2_M201_12 | G | C | C | G | G | T | T | T | A | T | T | T | T | T | G | G | T | G | G |
| FS2_M202_17 | G | C | C | G | G | T | T | T | A | T | T | T | T | T | G | G | T | G | G |

**Table S7** Chloroplast SNP Genotypes in 75 *Pinus taeda* Individuals (continued).

| **Sample** | **23167** | **23593** | **50842** | **50999** | **98314** | **101076** | **101085** | **101094** | **101099** | **101101** | **101103** | **101112** | **101121** | **101130** | **101139** | **101148** | **101157** | **101184** | **119153** |
| --- | --- | --- | --- | --- | --- | --- | --- | --- | --- | --- | --- | --- | --- | --- | --- | --- | --- | --- | --- |
| FS2_M243_22 | G | C | C | G | G | T | T | T | A | T | T | T | T | T | G | G | T | G | G |
| FS2_M259_27 | G | C | C | G | G | T | T | T | A | T | T | T | T | T | G | G | T | G | G |
| FS2_M288_32 | G | C | G | G | G | T | T | T | A | T | T | T | T | T | G | G | T | G | G |
| FS2_MW16_37 | A | A | T | A | G | T | T | T | A | T | G | T | G | T | T | G | T | T | G |
| FS2_MP100_42 | G | C | C | G | G | T | T | T | A | T | T | T | T | T | G | G | T | G | G |
| W03 | A | A | T | A | G | T | T | T | A | T | G | T | G | T | T | G | T | T | G |
| FW03_M014_3 | A | A | T | A | G | T | T | T | A | T | G | T | G | T | T | G | T | T | G |
| FW03_M017_8 | G | C | C | G | G | T | T | T | A | T | T | T | T | T | G | G | T | T | G |
| FW03_M201_13 | G | C | C | G | T | G | G | T | A | T | G | G | T | G | G | G | T | T | T |
| FW03_M202_18 | G | C | C | G | T | G | G | T | A | T | G | G | T | G | G | G | T | T | T |
| FW03_M243_23 | G | C | C | G | T | G | G | T | A | T | G | G | T | G | G | G | T | T | T |
| FW03_M259_28 | G | C | C | G | T | G | G | T | A | T | G | G | T | G | G | G | T | T | T |
| FW03_M288_33 | G | C | C | G | T | G | G | T | A | T | G | G | T | G | G | G | T | T | T |
| FW03_MW16_38 | G | C | C | G | G | T | T | T | A | T | T | T | G | G | G | G | T | T | G |
| FW03_MP100_43 | G | C | C | G | T | T | T | T | A | T | T | T | T | T | T | T | G | T | T |
